# Impact of Ultrasonic Scaling on Microleakage in Lithium Disilicate Crowns Luted With Different Resin Cements

**DOI:** 10.1155/ijod/9215253

**Published:** 2025-10-16

**Authors:** Waleed AL-Mutairi, Marwa Eltayeb I. Elagra

**Affiliations:** Department of Prosthodontics, Riyadh Elm University, Riyadh, Saudi Arabia

**Keywords:** E-max CAD/CAM, lithium disilicate crown, microleakage, self-adhesive cements, self-etching, total etching, ultrasonic scaler

## Abstract

**Aim:**

To assess the impact of ultrasonic scaling on the microleakage at the lithium disilicate crown margins with three different techniques of resin cements.

**Materials and Methods:**

Forty-eight human teeth were fixed in acrylic resin mold within 2 mm away from the cementoenamel junction. A 1.5 mm occlusal and axial surface preparation with 12° taper and heavy chamfer of 1 mm thickness was performed. E-max CAD/CAM crown was fabricated for each sample. Samples were categorized according to the cement used into: Group 1 (*n* = 16) cemented with self-etching, Group 2 (*n* = 16) cemented with self-adhesive, and Group 3 (*n* = 16) cemented with total-etch cement. Each group was subsequently divided into two equal subgroups: one undergoing water aging (10,000 thermocycles) without ultrasonic scaling (*n* = 8) and the other subjected to both water aging (10,000 thermocycles) and ultrasonic scaling. All samples were then examined under microscopes (*n* = 8).

**Result:**

The overall comparison of microleakage among the three cements was not statistically significant (*p*=0.49). The highest microleakage was observed in total-etching cement (5.69), while the lowest was in self-etching cement without scaling (0.88). No significant difference was found between nonscaling and scaling within the same cement group: Group 1 (*p*=0.92), Group 2 (*p*=0.64), and Group 3 (*p*=0.89).

**Conclusion:**

No statistically significant differences were found in microleakage around lithium disilicate crowns cemented with various types of resin cements, nor between groups subjected to ultrasonic scaling and those that were not.

## 1. Introduction

The growing demand from patients for more natural-looking restorations has driven the advancement of new all-ceramic materials. All ceramic crowns have enhanced mechanical properties that warrant strength, limited technical drawbacks, and prolonged clinical life [1].

In recent years, restorative dentistry has witnessed the availability of advanced materials. Among these restorative materials, lithium disilicate is a noteworthy addition. This material has excellent esthetics, strength, durability, biocompatibility, and marginal integrity [2]. Moreover, its superior abrasion resistance makes it an ideal all-ceramic material for crowning the teeth. Compared to zirconia, the translucency of lithium disilicate crowns is higher [3]. Using transparent cement with a higher degree of translucency may result in a more natural-looking repair [4].

Marginal adaptation is critical in fixed prosthodontics. One of the most critical technical aspects of a restoration's long-term performance seems to be a solid marginal fit [5]. The rate of cement disintegration and microleakage may lead to pulpal irritation. Additionally, it can contribute to the onset of periodontal disease [6]. Marginal misfit exposes the luting cement to oral fluids, leading to its dissolution and disintegration, which results in microleakage [7].

In recent years, the trend toward using resin-luting materials has increased dramatically. In contrast to traditional glass ionomer (GIC), zinc phosphate, polycarboxylate, and resin-modified GIC–based luting cements, composite resin cement offers improved esthetics, low solubility, high strength, and strong adhesion [8]. For cementing all-ceramic crowns, resin luting agents are recommended due to their ability to enhance the strength of the restoration [9]. There are three subtypes of resin-luting cement: self-etching primer, total-etch, and self-adhesive.

Total-etch systems (light-cured, dual-cured, or self-cured) involve three fundamental phases: Initially, the tooth is treated with an acid etch, followed by cleaning, and then, it is gently dried. The bonding agent is then applied and cured. Either a light-cured bonding agent or a two-part self-curing system can be used. Next, the cement is mixed, applied to the restoration, positioned, cleaned, and cured.

In self-etching systems, a self-etching primer substitutes the acid etching and bonding steps. In contrast, self-adhesive systems eliminate separate etching and bonding steps, resulting in a simplified procedure [10]. Additionally, a study assessed the degree of microleakage at the interface between the tooth and restoration in Class V composite resin restorations using two self-etch systems (Adper SE Plus and Adper Easy One), one total-etch system (Adper Single Bond), and universal bonding agents, employing the dye penetration method. The findings indicated that the one-step self-etch agent exhibited less microleakage at the occlusal margin compared to the total etch and universal adhesive systems [11]. However, a study conducted by Geerts et al. [12] assessed the bonding performance of various dental adhesives, including two self-etch and two etch-and-rinse adhesives, following thermocycling. It concluded that a three-step etch-and-rinse adhesive demonstrated significantly less microleakage compared to other adhesive systems and could be considered a reference adhesive.

Scaling and root planning is an important technique for controlling biofilm formation and maintaining periodontal health [13]. Sonic and ultrasonic scalers are conventional instruments that remove plaque and calculus from the tooth surface. Sonic scalers operate at frequencies between 3000 and 8000 cycles per second (Cps). In contrast, ultrasonic scalers are categorized into two main types: magnetostrictive, which vibrates between 18,000 and 45,000 Cps and features an elliptical vibration pattern active on all sides of the tip, and piezoelectric, which operates at 25,000 to 50,000 Cps with a linear vibration pattern that activates only two edges of the tip [14]. The magnetostrictive scaler's energy dispersion makes all sides effective, with the tip producing the most vibrations, potentially causing patient discomfort and damage. The piezoelectric scaler, on the other hand, requires less water to control heat, providing a distinct advantage [15].

Previous studies have examined the impact of ultrasonic scaling on microleakage around zirconia crowns cemented with various resin-based cements. These investigations generally reported that ultrasonic instrumentation did not significantly increase microleakage at the crown margins and that different types of resin cements exhibited varying levels of resistance to such procedures [9, 16]. However, despite these findings, limited evidence exists regarding the influence of ultrasonic scaling on microleakage around lithium disilicate crowns. This gap in the literature underscores the need for further research to evaluate the performance of resin cements in this context and to provide guidance for clinical decision-making.

### 1.1. Aim of the Study

The purpose of this in vitro study was to evaluate the effect of ultrasonic scaling on microleakage around lithium disilicate crown margins cemented with three different types of resin luting cement (i.e., self-adhesive [Relyx U200 Automix], self-etch [Olicem SE], and total-etch [Relyx Veneer cement]).

The null hypotheses tested in this study were that there is no statistically significant difference in the microleakage of lithium disilicate crowns cemented with self-etch, self-adhesive, or total-etch resin cements and that ultrasonic scaling does not have a statistically significant effect on the microleakage of lithium disilicate crowns.

## 2. Materials and Methods

### 2.1. Sample Preparation

In this study, 48 extracted human maxillary premolars were used. The teeth were collected from an orthodontic clinic, where they had been extracted as part of routine orthodontic treatment. Only sound teeth free of caries, cracks, fracture lines, restorations, and other structural defects were included. The average crown dimensions were approximately 6–7 mm in both cervico-occlusal and mesiodistal directions. Each tooth was examined using a sharp explorer to confirm its suitability, and any teeth not meeting the inclusion criteria were excluded. To prevent microbial growth, the selected teeth were stored in 0.5% Chloramine T solution at 4°C. Ethical approval for the use of extracted teeth in this study was obtained from the institutional review board.

All tooth preparations were conducted by a highly experienced operator. The teeth were securely fixed in acrylic resin mold, with about 2 mm of space from the cementoenamel junction. A consistent cervical margin thickness of 1.0 mm was maintained around the tooth, and a 0.5 mm deep chamfer was created coronal to the cementoenamel junction.

Utilizing a CAD/CAM device (CEREC Omnicam, Sirona, Germany), the entire teeth underwent digital scanning. The full-anatomic crown design included walls that were 1.5 mm thick in the contact area, 1.0 mm at the apex, and 1.5 mm in the occlusal region. A specified cement thickness of 50 mm, starting 1.0 mm beyond the edge, was adhered to for all crowns. Subsequently, the crowns were milled from lithium disilicate ceramic blocks (IPS e.max CAD, size C14, color HT A2, Ivoclar Vivadent, Schaan, Liechtenstein) using the CEREC milling machine (Bluecam, Bensheim, Germany). The sintering cycle reached a temperature of 840°C, followed by glazing and the crystallization process.

The E-max crowns were meticulously positioned on the correspondingly prepared teeth to ensure an optimal fit. A single administrator conducted a precise marginal fit assessment using an explorer and visual inspection. Following this evaluation, sample cleaning was executed using a prophy brush filled with water and pumice flour.

### 2.2. Grouping

Teeth featuring lithium disilicate crowns were categorized into three distinct study groups. Group 1 (*n* = 16) underwent cementation with self-etching (Olicem SE), Group 2 (*n* = 16) with self-adhesive (3M ESPE Relyx U200 Automix), and Group 3 (*n* = 16) with total-etch (3 M-ESPE Relyx Veneer cement). Each study group was further subdivided into two subgroups (*n* = 8): one subjected to water aging (10,000 thermocycles) without ultrasonic scaling and the other undergoing both water aging (10,000 thermocycles) and ultrasonic scaling.

### 2.3. Cementation

The cementation protocols followed the manufacturers' recommendations and were adapted to the specific type of resin cement used. For all groups, the internal surface of each crown was treated with 5% hydrofluoric acid for 20 s, rinsed, air-dried, and then, coated with a silane coupling agent for 60 s before light air-drying. Tooth surfaces were cleaned using a pumice slurry, rinsed, and lightly dried. For the total-etch group, 37% phosphoric acid was applied to the tooth for 15 s, rinsed, and followed by two coats of an adhesive bonding agent, then, air-dried. For the self-etch group, a self-etching adhesive was rubbed onto the tooth surface for 20 s and light cured for 20 s. The self-adhesive group did not require additional tooth surface treatment. Cement was applied to the crown in all groups, and the crown was seated with gentle pressure under a static load holder to ensure uniform pressure. Initial light curing was done for 2 s to facilitate the removal of excess cement, followed by final light polymerization for 30 s on each crown margin using a CU-1000 curing unit.

### 2.4. Ultrasonic Scaling and Thermocycling

Ultrasonic scaling was performed using a piezoelectric device (Woodpecker UDS-P LED) with a scaling tip (Woodpecker G4 scaler tip) at full power, utilizing distilled water. The scaling procedure involved moving the lateral side of the tip along the crown–dentin interface for 60 s on each side under moderate hand pressure. Additionally, 10,000 cycles of thermocycling, alternating between a cold bath (5°C for 15 s) and a hot bath (55°C for 15 s) with a 10 s water dripping time at every temperature, were executed.

### 2.5. Microleakage Test

**Figure 1:**
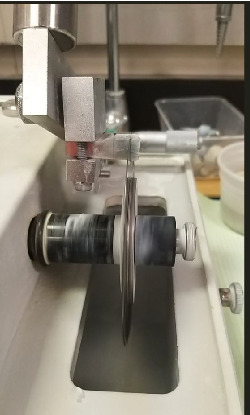
Sample sectioning utilizing HIROX Digital Microscope.

Root surfaces of all teeth were shielded with nail varnish, leaving a 1-mm margin around the cemented area to prevent methylene blue dye penetration, especially in the apical foramen. After a 24 h soak in a 2% basic methylene blue dye solution, samples were thoroughly washed in water. The specimens were then embedded in cold-curing orthodontic acrylic resin (Caulk, DENTSPLY, York, PA) and buccolingually sectioned through the crown center using a precision diamond saw (Isomet 5000; Buehler Ltd., IL, USA) at 1800 rpm under water cooling (Figure 1).

**Figure 2:**
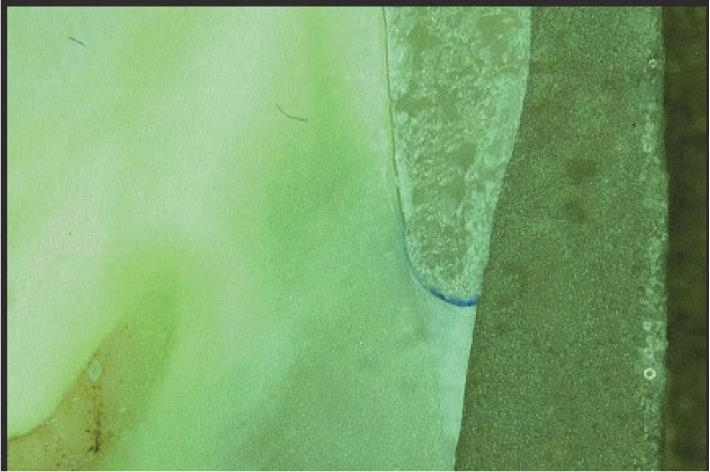
Sample under 50x magnification using a HIROX Digital Stereomicroscope (KH-7700).

Dye penetration was assessed at a magnification of 50x using a stereomicroscope (HIROX, Digital Microscope KH-7700) to observe from the external crown surface until no blue dye was visible (Figure 2). Utilizing image analysis software (Analyze Starter, Soft Image System, Germany), the extent of dye penetration in both buccal and lingual sections across all study groups was precisely computed. The degree of dye penetration was recorded by one operator, using internal image analysis software (Measurement Tool, HIROX), and measured from the external crown surface to the point where no blue dye was visible at a magnification of 50. To measure microleakage, the percentage was calculated by dividing the length of dye penetration by the distance from the outer margin to the axial occlusal line angle. Additionally, the extent of dye penetration was categorized on a scale from 0 to 6, where 0 indicates no dye penetration and 6 indicates dye penetration along all the axial walls up to the occlusal edge, following the classification by Ebadian et al. [17].

### 2.6. Result

Nine out of 48 samples showed microleakage. Dye penetrated, one third of the chamfer in two samples, two thirds of the chamfer in two samples, the entire chamfer in two samples, and one third of axial wall in three samples. The highest number of microleakage and the greatest depth of penetration was demonstrated in samples cemented with total etch cement (Table 1).

**Figure 3:**
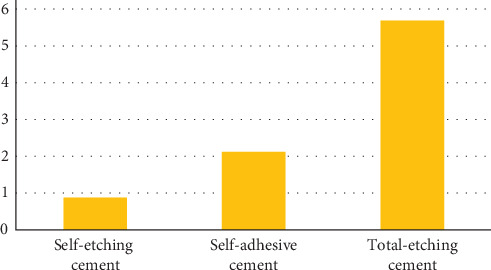
Comparison of microleakage across all cements (combined scaling and no scaling).

Microleakage in whole samples ranged between 4% and 27%. The overall (combination of scaling and nonscaling samples) comparison of microleakage between all the three cements was statistically nonsignificant (*p*=0.49). The highest and lowest microleakage was observed in total-etching cement (5.69) and self-etching cement (0.88), respectively (Figure 3).

**Figure 4:**
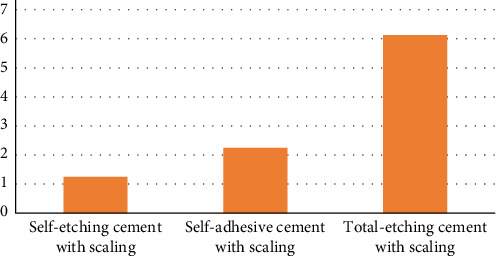
Microleakage comparison among all cements after ultrasonic scaling.

The Kruskal–Wallis test showed a statistically nonsignificant difference (*p*=0.71) of microleakage among ultrasonic scaling groups of all three cements. The highest and lowest microleakage was observed in total-etching cement (6.13) and self-etching cement (1.25), respectively (Figure 4).

**Figure 5:**
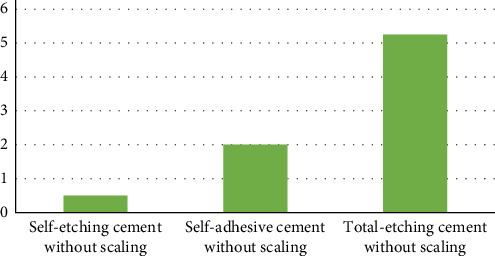
Microleakage comparison among all cements without ultrasonic scaling.

The Kruskal–Wallis test showed statistically nonsignificant (*p*=0.64) difference of microleakage among nonscaling groups of all three cements. The highest and lowest microleakage was observed in total-etching cement without scaling (5.25) and self-etching cement without scaling (0.5), respectively (Figure 5).

**Figure 6:**
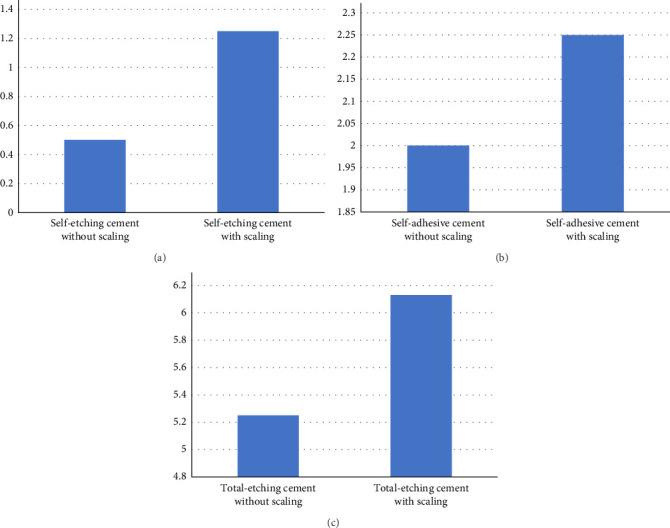
Microleakage comparison with and without scaling: (a) self-etching cement, (b) self-adhesive cement, and (c) total-etching cement.

The microleakage was greater in self-etching cement with scaling (1.25) than without scaling (0.5). This difference was statistically nonsignificant (*p*=0.92; Figure 6a).

The difference in microleakage between self-adhesive cement with and without scaling groups was statistically nonsignificant (*p*=0.64). The microleakage was marginally more in self-adhesive cement with scaling (2.25) than without scaling (2.0; Figure 6b).

The total-etching cement showed a statistically nonsignificant difference (*p*=0.89) of microleakage between scaling (6.13) and nonscaling groups (5.25; Figure 6c).

## 3. Discussion

Limited data exists on the impact of ultrasonic scaling on the microleakage of lithium disilicate crowns luted with resin cement. Therefore, this laboratory study aimed to investigate the effects of ultrasonic scaling on microleakage around the margins of lithium disilicate crowns cemented with three different types of resin luting cement: self-adhesive (RelyX U200 Automix), self-etch (Olicem SE), and total-etch (RelyX Veneer Cement).

Although in this study the total etching samples had the highest microleakage mean value followed by self-adhesive, and the lowest mean value was demonstrated in the self-etching samples, the difference was statistically not significant. Therefore, the first null hypothesis, there was no difference in microleakage using various resin-luting cements, was accepted.

The higher mean microleakage observed in the total-etching group could be attributed to the light-curing process through the 1.5 mm thick ceramic crowns. The thickness and shade of ceramics are known to reduce light transmission, potentially compromising the polymerization and bonding quality of light-cured resin cements [18]. This may explain why light cured total-etching cements, which rely heavily on adequate light activation, showed relatively higher microleakage in this study.

This finding is supported by Gupta et al. [11], who demonstrated that one-step self-etch adhesives exhibited less microleakage than total-etching techniques, likely due to improved adaptation and simplified application. Similarly, Cal et al. [19] evaluated self-adhesive and etch-and-rinse resin cements and found no statistically significant difference in microleakage between them at the cervical margin. The authors explained that the multifunctional phosphoric acid methacrylate in self-adhesive cements allows for both micromechanical and chemical bonding, including hydrogen bonding, dipole interactions, and complex formation with calcium ions—factors which contribute to a reliable seal even without separate etching and bonding steps.

In contrast, El Sayed et al. [20] and Geerts et al. [12] both advocated for the superiority of etch-and-rinse systems, noting their lower microleakage and better bonding performance, especially when using a three-step approach. However, even though the total-etch group in the present study showed the highest microleakage values numerically, the differences among groups were not statistically significant, potentially due to variations in bonding protocols or the specific adhesive systems used.

These findings are partially consistent with those reported by Sengar et al. [21], who evaluated microleakage among different adhesive strategies and found no significant difference between self-etch and total-etch groups, but a significant difference between total-etch and self-adhesive cements. They attributed the higher leakage in self-adhesive systems to their increased water absorption, which may impair bonding integrity overtime.

Scaling and nonscaling groups had approximately the same mean value of microleakage, and the difference was statistically not significant. Therefore, the second null hypothesis—there was no difference between microleakage observed in lithium disilicate with or without ultrasonic scaling—was accepted.

This result aligns with Chang et al. [16], who reported that ultrasonic scaling did not significantly affect microleakage in zirconia crowns cemented with self-adhesive or resin-modified glass ionomer cements. Similarly, Rohani et al. [22] found no significant difference in microleakage between ultrasonic and manual instrumentation on various restorative materials, regardless of whether margins were on enamel or cementum.

One explanation for the absence of increased microleakage after ultrasonic scaling is that all crown margins in this study were located on enamel. Previous studies have shown that ultrasonic instrumentation tends to increase microleakage at dentin or cementum interfaces, but has little to no effect at enamel margins [16, 23]. Additionally, Angerame et al. [24] found that instrumentation on enamel margins resulted in a larger marginal gap, but reduced microleakage, potentially due to debris from instrumentation filling the gaps and preventing dye penetration.

Ultrasonic scaling was also hypothesized to create marginal microleakage from cement roughening and removal. However, the margins in this study were well-adapted with no visible marginal gaps. Given that the diameter of the ultrasonic scaler tip is approximately 270 μm, it is unlikely that it could have accessed or dislodged cement from such closely adapted interfaces. Nevertheless, in clinical situations where margins are open or poorly adapted, the scaler tip may be able to remove cement or create ledges that facilitate vibrational damage [16]. Therefore, the results of this study may not be applicable in cases with existing marginal discrepancies.

However, in contrast to our results, Azer [9] found significantly greater microleakage in zirconia crowns luted with self-adhesive resin cements after ultrasonic scaling compared to those luted with self-etch adhesive systems. They suggested that the self-adhesive cement was more vulnerable to degradation under ultrasonic stress, possibly due to weaker bonding and lower resistance to mechanical disruption.

The existing shortage in availability of self-etching, self-adhesive, and total-etching resin cements with the same curing technique might contribute to varying proportions of microleakage. A further limitation was that dye measurements were only taken in the line section, not across the whole cementation margin, which may have contributed to the decrease in the microleakage rate. While microleakage tests are widely used, relying solely on a dye penetration test should not be considered conclusive. Numerous reviews suggest that clinical outcomes such as marginal discoloration, hypersensitivity, and secondary caries do not directly correlate with dye penetration. Additionally, this study has limitations, as the test methodology does not fully replicate the conditions of the oral cavity, such as varying amounts and pH of saliva, occlusal forces, and the presence of *Lactobacillus* and *Streptococcus mutans* [25].

## 4. Conclusion

• Among the tested resin cements, the total-etch cement demonstrated the highest percentage of microleakage. However, the differences in microleakage among the three types of cement were not statistically significant.
• There was no significant difference in microleakage between scaling and nonscaling groups.
• There was no significant difference in microleakage between nonscaling and scaling groups with self-etching, self-adhesive, and total-etching cements.

## Figures and Tables

**Table 1:** Microleakage observations for different cements.

Cement type	Samples	No microleakage	Microleakage	Percentage of microleakage
1- Self-etching cement without scaling	8	7	1	4%
2- Self-etching cement with scaling	8	7	1	10%
3- Self-adhesive cement without scaling	8	7	1	16%
4- Self-adhesive cement with scaling	8	6	2	6%, 12%
5- Total-etching cement without scaling	8	6	2	19%, 23%
6- Total-etching cement with scaling	8	6	2	22%, 27%

*Note:* The percentages for microleakage in some cases are presented as separate values for individual samples.

## Data Availability

The datasets analyzed during the current study are available from the corresponding author upon reasonable request.
